# Plasma 25-hydroxyvitamin D concentrations, vitamin D deficiency and mortality in community-dwelling Japanese adults

**DOI:** 10.1017/S0007114525105308

**Published:** 2025-10-28

**Authors:** Kaori Kitamura, Yumi Watanabe, Keiko Kabasawa, Toshiko Saito, Akemi Takahashi, Ryosaku Kobayashi, Rieko Oshiki, Kei Watanabe, Ribeka Takachi, Shoichiro Tsugane, Kazutoshi Nakamura

**Affiliations:** 1 Division of Preventive Medicine, Niigata University Graduate School of Medical and Dental Sciences, Niigata, Japan; 2 Department of Health Promotion Medicine, Niigata University Graduate School of Medical and Dental Sciences, Niigata, Japan; 3 Department of Rehabilitation, Niigata University of Rehabilitation, Niigata, Japan; 4 Division of Orthopedic Surgery, Niigata University Graduate School of Medical and Dental Sciences, Niigata, Japan; 5 Department of Food Science and Nutrition, Nara Women’s University Graduate School of Humanities and Sciences, Nara, Japan; 6 International University of Health and Welfare Graduate School of Public Health, Tokyo, Japan

**Keywords:** Cohort study, Mortality, Season, Sex, Vitamin D

## Abstract

Blood 25-hydroxyvitamin D (25(OH)D) concentrations vary considerably by season and sex. The present study aimed to determine associations between vitamin D deficiency and mortality in Japanese adults and identify risk thresholds according to 25(OH)D concentrations. This was a cohort study with an 11-year follow-up. Participants were 8285 community-dwelling Japanese adults aged 40–74 years. Plasma 25(OH)D concentrations were measured by chemiluminescent immunoassay at baseline and divided into quintiles for each of the subgroups stratified by season and sex (denoted as season- and sex-stratified quintiles). The main outcome was all-cause mortality. Hazard ratios (HR) were calculated using a Cox proportional hazards model. Mean age and 25(OH)D concentration were 59·9 years (sd = 9·1) and 50·1 nmol/l (sd = 18·1), respectively. Lower season- and sex-stratified quintiles were associated with higher hazards of all-cause mortality (*P*_for trend_ = 0·0015), with the first quintile (median = 28·2 nmol/l) having a higher HR (HR = 1·46, 95 % CI, 1·13, 1·88) than the highest quintile (reference). When crude quintiles were used, the overall association was similar (*P*_for trend_ = 0·0027), with the first (median = 28·0 nmol/l) and second (median = 39·7 nmol/l) quintiles having higher HR (HR = 1·40, 95 % CI, 1·06, 1·85 and 1·38, 95 % CI, 1·07, 1·77, respectively) than the reference. The risk threshold difference was estimated to be approximately 10 nmol/l. In conclusion, low blood 25(OH)D concentrations are associated with high mortality risk. Crude blood 25(OH)D concentration may modulate the estimated risk threshold for vitamin D deficiency associated with mortality.

Vitamin D plays an important role in regulating cell proliferation/differentiation, the immune system, cardiovascular protection and bone metabolism^(1,2)^. Consistent with this, low levels of vitamin D in the body (i.e. vitamin D deficiency) are considered detrimental to many aspects of health. When vitamin D deficiency is defined as blood 25-hydroxyvitamin D (25(OH)D) concentrations < 50 nmol/l, the prevalence of vitamin D deficiency is reported to be 24–40 % in European and North American countries, and the prevalence may be even higher in other populations^(3)^.

Meta-analyses have found that vitamin D deficiency is associated with increased all-cause mortality, and this association is observed consistently across various general populations worldwide^(4,5)^. However, reports on the threshold of blood 25(OH)D concentrations, an appropriate biomarker of vitamin D status, at which all-cause mortality begins to increase are inconsistent. Two meta-analyses published in 2012^(4)^ and 2014^(6)^ suggested < 75 nmol/l of blood 25(OH)D increases all-cause mortality risk. However, more recently published studies suggested lower thresholds, such as 35^(7)^, 40^(8)^, 50^(9)^, 50^(10)^, 55^(11)^, 45–60^(12)^ and 65 nmol/l^(13)^. These differences may be attributed to several factors, such as region, demographics, lifestyle, environmental factors and ethnicity^(5,14)^.

The circannual change in sunlight exposure is a major factor influencing seasonal variation in blood 25(OH)D concentrations; concentrations are typically highest in summer and lowest in winter^(15)^. Previous cohort studies have introduced season as a covariate when determining the threshold of blood 25(OH)D concentrations for mortality risk, but they rarely accounted for annual variation in 25(OH)D concentrations. Moreover, sex differences in blood 25(OH)D concentrations are considerable. According to a Japanese study, the prevalence of vitamin D deficiency in women is approximately twice that of men^(16)^, and thus sex differences may not be fully accounted for by statistical adjustments.

The Murakami cohort study of vitamin D (established in 2011, *n* 8498) is a population-based cohort study to determine the effect of vitamin D on age-related diseases in middle-aged and older people^(16,17)^. In the present study, plasma 25(OH)D concentrations were stratified by month (of blood collection) and sex to classify vitamin D level groups, allowing for season- and sex-stratified 25(OH)D groupings. We aimed to determine associations between vitamin D deficiency and mortality and identify risk thresholds in middle-aged and older individuals. Specifically, we examined risk thresholds of 25(OH)D concentrations for all-cause mortality by comparing season- and sex-stratified 25(OH)D groups with crude 25(OH)D groups.

## Methods

### Design and participants

This cohort study had an 11-year follow-up component. The Murakami cohort study targeted all 34 802 community-dwelling individuals aged 40–74 years residing in Murakami City, Sekikawa Village and Awashimaura Village in Japan, and 14 364 participated in the study. Of these, 8497 provided plasma samples. We excluded the following individuals: (1) 176 who had missing values for education, BMI or lifestyle information and 36 whose BMI values were outliers. The final population for statistical analysis included 8285 individuals. This study was conducted according to the guidelines laid down in the Declaration of Helsinki, and all procedures involving human subjects/patients were approved by the Ethics Committee of Niigata University (Nos. 452 and 1324). Written informed consent was obtained from all participants.

### Baseline survey

The baseline survey of the Murakami cohort study was conducted between 2011 and 2013. We used a self-administered questionnaire to obtain information on demographics, lifestyles, body size (height and weight) and disease history, including cancer, stroke, heart disease (myocardial infarction, angina, atrial fibrillation and/or heart failure), hypertension and diabetes. Codes for marital status, education level, occupation, smoking, alcohol consumption and disease histories are shown in online Supplementary Table 1. Total physical activity (PA) levels were assessed using the metabolic equivalent of task index (metabolic equivalent of task-h/d), which was calculated by multiplying the intensity of each activity by the number of hours per day using the Japan Public Health Center-based prospective study – physical activity questionnaire^(18)^. Intensity of activities used in this calculation is also shown in online Supplementary Table 1. BMI was calculated as weight (kg) divided by the square of height (m^2^). Details of the baseline survey have been published elsewhere^(17)^.

### Vitamin D measurements

Non-fasting blood specimens were collected, and plasma was obtained. Plasma specimens were stored at –80°C until biochemical analysis. Plasma 25(OH)D concentrations were measured by chemiluminescent immunoassay using the Liaison® 25OH Vitamin D Total Assay (DiaSorin Inc.; Stillwater, MN, USA). Intra- and inter-assay CV values were 3·2–8·1 % and 6·9–12·7 %, respectively. Details of collection and handling of blood specimens have been reported previously^(16,17)^.

### Mortality during follow-up period

Information on dates of death and relocations up until 31 March 2024 were obtained from death and residency registration records in accordance with the Basic Residential Registry Law and the Family Registry Law. Information on causes of death was available up until 31 December 2022.

### Statistical analysis

Median and interquartile range and numbers (and percentages) were used to characterise continuous variables and dummy variables, respectively. *P*_for trend_ values for linear associations between 25(OH)D quintiles and participant characteristics were calculated using ordinal logistic regression analysis. Seasons were classified into five categories based on mean monthly plasma 25(OH)D concentrations at baseline^(17)^, as follows: (1) March and April (mean 25(OH)D, 42·6–42·8 nmol/l); (2) February and May (45·7–47·3 nmol/l); (3) June (49·0 nmol/l); (4) July, October, November, December and January (51·9–53·3 nmol/l) and (5) August and September (58·3–58·8 nmol/l). These categories were assigned numerical values and referred to as the ‘season score’. To account for seasonal- and sex-related variations in 25(OH)D concentrations, plasma 25(OH)D concentrations were divided into quintiles within each of the ten subgroups defined by season (five groups) and sex (two groups). These quintiles are referred to as ‘season- and sex-stratified quintiles’ herein. Hazard ratios (HR) for mortality according to quintiles were calculated using a Cox proportional hazards model. Covariates for the multivariate model were age, marital status (dummy variable), education, occupation (dummy variable), BMI, total PA, smoking, alcohol consumption and disease history for the season- and sex-stratified quintile model and season score, sex, age, marital status, education, occupation, BMI, total PA, smoking, alcohol consumption and disease history for the crude quintile model. Cubic spline curves with five knots were drawn to illustrate the multivariable-adjusted association between (a) season- and sex-stratified quintiles and (b) crude quintiles of plasma 25(OH)D concentrations and mortality hazard. Statistical analysis was performed using SAS statistical software (release 9.4, SAS Institute Inc.). *P* < 0·05 was considered statistically significant.

## Results

**Figure 1.**
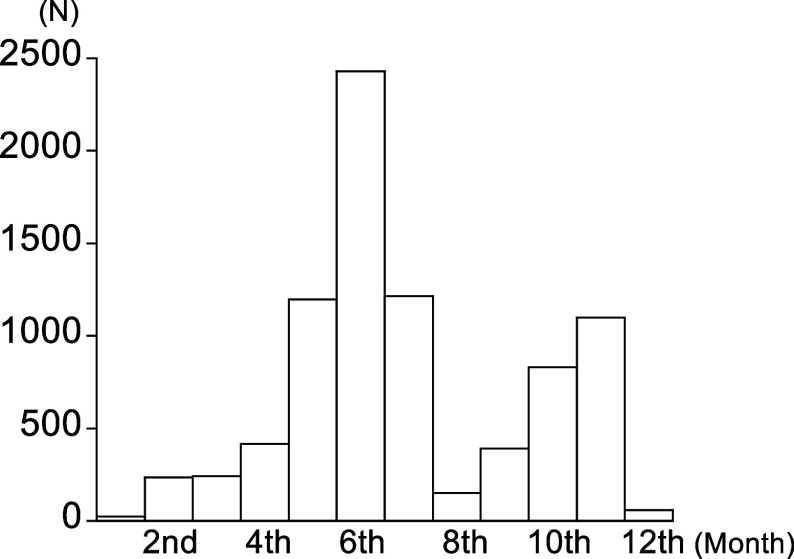
Monthly distribution of blood collection.

Mean age, 25(OH)D concentration and follow-up period of participants were 59·9 years (sd, 9·1), 50·1 nmol/l (sd, 18·1) and 11·4 years (sd, 1·5), respectively. The number of participants by month of blood collection is shown in Figure 1, which shows that fewer samples were collected during the winter months. The prevalence of low plasma 25(OH)D concentrations (defined as < 50 nmol/l) according to season score and sex at baseline is shown in online Supplementary Table 2. Agreement between the season- and sex-stratified and crude quintiles of 25(OH)D concentrations is shown in online Supplementary Table 3. The weighted κ coefficient was 0·72 (95 % CI: 0·71, 0·73). The number of deaths during the follow-up period was 641, including twenty participants in their 40s, 75 in their 50s, 258 in their 60s and 288 in their 70s. Cumulative all-cause mortality rates as determined by the Kaplan–Meier method stratified by crude quintiles of 25(OH)D concentrations are shown in online Supplementary Figure 1.

Participant characteristics according to season- and sex-stratified and crude quintiles of plasma 25(OH)D concentrations are shown in Table 1. Season- and sex-stratified quintiles were significantly, positively associated with age, total PA, being married, having a manual job, being a current drinker and having a history of hypertension and inversely associated with education level and being a current smoker. Crude quintiles were significantly, positively associated with age, BMI, total PA, being male, being married, having a manual job and having a history of heart disease, stroke or hypertension.

**Table 1.** Participant characteristics according to season- and sex-stratified and crude quintiles of plasma 25-hydroxyvitamin D (25(OH)D) concentrations

	Season- and sex-stratified 25(OH)D quintiles										*P* for trend
Characteristics	Quintile 1		Quintile 2		Quintile 3		Quintile 4		Quintile 5		
*n*	1664		1661		1663		1658		1639		
	Median	Minimum, 25th percentile, 75th percentile, maximum	Median	Minimum, 25th percentile, 75th percentile, maximum	Median	Minimum, 25th percentile, 75th percentile, maximum	Median	Minimum, 25th percentile, 75th percentile, maximum	Median	Minimum, 25th percentile, 75th percentile, maximum	
25(OH)D (nmol/l)	28·2	8·5, 22·9, 33·2, 53·9	39·4	24·8, 35·4, 45·4, 63·6	47·4	32·4, 43·7, 55·4, 72·6	56·4	40·2, 51·9, 64·9, 83·4	72·9	52·7, 65·1, 82·6, 144·3	<0·0001
	Median	IQR	Median	IQR	Median	IQR	Median	IQR	Median	IQR	
Follow-up years	11·7	11·4–11·8	11·7	11·4–11·8	11·7	11·4–11·8	11·7	11·4–11·8	11·7	11·4–11·8	
Age (years)	55·0	48–62	60·0	52–67	62·0	55–68	63·0	56–69	63·0	58–69	<0·0001
BMI (kg/m^2^)	22·7	20·7–24·9	23·0	21·1–25·2	23·0	21·2–25·1	23·0	21·2–24·8	22·5	20·8–24·4	0·1177
Total PA (MET-h/d)	40·2	36·2–49·4	42·3	37·1–51·5	43·1	37·8–53·1	44·0	38·2–53·2	45·0	38·5–55·8	<0·0001
	*n*	%	*n*	%	*n*	%	*n*	%	*n*	%	
Men	724	43·5 %	722	43·5 %	730	43·9 %	725	43·7 %	715	43·6 %	0·8983
Married	1266	76·1 %	1366	82·2 %	1381	83·0 %	1402	84·6 %	1374	83·8 %	<0·0001
University	124	7·5 %	101	6·1 %	94	5·7 %	95	5·7 %	67	4·1 %	0·0001
Manual job	341	20·5 %	356	21·4 %	352	21·2 %	360	21·7 %	411	25·1 %	0·0032
Current smoker	374	22·5 %	260	15·7 %	222	13·3 %	207	12·5 %	216	13·2 %	<0·0001
Current drinker	885	53·2 %	891	53·6 %	949	57·1 %	967	58·3 %	973	59·4 %	<0·0001
History of											
Cancer	96	5·8 %	94	5·7 %	108	6·5 %	102	6·2 %	94	5·7 %	0·8129
Heart disease	54	3·2 %	85	5·1 %	90	5·4 %	83	5·0 %	80	4·9 %	0·0667
Stroke	29	1·7 %	29	1·7 %	29	1·7 %	37	2·2 %	40	2·4 %	0·0776
Hypertension	281	16·9 %	327	19·7 %	357	21·5 %	391	23·6 %	368	22·5 %	<0·0001
Diabetes	119	7·2 %	130	7·8 %	123	7·4 %	120	7·2 %	100	6·1 %	0·1925

	Crude 25(OH)D (nmol/l) quintiles^*^										*P* for trend
Characteristics	Quintile 1		Quintile 2		Quintile 3		Quintile 4		Quintile 5		
*n*	1693		1594		1716		1640		1642		
	Median	Minimum, 25th percentile, 75th percentile, maximum	Median	Minimum, 25th percentile, 75th percentile, maximum	Median	Minimum, 25th percentile, 75th percentile, maximum	Median	Minimum, 25th percentile, 75th percentile, maximum	Median	Minimum, 25th percentile, 75th percentile, maximum	
25(OH)D (nmol/l)	28·0	8·5, 23·0, 31·5, 34·7	39·7	34·9, 37·4, 41·9, 43·9	48·7	44·2, 46·4, 50·9, 53·4	58·9	53·7, 56·2, 62·2, 65·1	73·9	65·4, 69·1, 82·6, 144·3	<0·0001
	Median	IQR	Median	IQR	Median	IQR	Median	IQR	Median	IQR	
Follow-up years	11·8	11·4–11·8	11·8	11·4–11·8	11·7	11·4–11·8	11·7	11·4–11·8	11·7	11·4–11·8	
Age (years)	56	48–63	60	53–67	62	55–68	63	56–69	63	58–69	<0·0001
BMI (kg/m^2^)	22·3	20·5–24·6	23·1	20·9–25·4	23·0	21·2–25·0	23·0	21·2–24·9	22·8	21·2–24·7	0·0046
Total PA (MET-h/d)	41·1	37·1–49·9	42·8	37·8–51·9	43·8	38·4–53·0	44·4	38·6–53·7	46·0	39·1–57·6	<0·0001
	*n*	%	*n*	%	*n*	%	*n*	%	*n*	%	
Men	427	25·2 %	537	33·7 %	663	38·6 %	866	52·8 %	1123	68·4 %	<0·0001
Married	1269	75·0 %	1322	82·9 %	1422	82·9 %	1373	83·7 %	1403	85·4 %	<0·0001
University	93	5·5 %	91	5·7 %	97	5·7 %	101	6·2 %	99	6·0 %	0·4010
Manual job	309	18·3 %	319	20·0 %	325	18·9 %	413	25·2 %	454	27·6 %	<0·0001
Current smoker	298	17·6 %	208	13·0 %	224	13·1 %	240	14·6 %	309	18·8 %	0·1551
Current drinker	758	44·8 %	804	50·4 %	922	53·7 %	992	60·5 %	1189	72·4 %	<0·0001
History of											
Cancer	87	5·1 %	88	5·5 %	116	6·8 %	100	6·1 %	103	6·3 %	0·1285
Heart disease	44	2·6 %	81	5·1 %	80	4·7 %	96	5·9 %	91	5·5 %	<0·0001
Stroke	27	1·6 %	28	1·8 %	29	1·7 %	29	1·8 %	51	3·1 %	0·0032
Hypertension	261	15·4 %	303	19·0 %	379	22·1 %	378	23·0 %	403	24·5 %	<0·0001
Diabetes	100	5·9 %	115	7·2 %	138	8·0 %	123	7·5 %	116	7·1 %	0·2002

PA, physical activity; MET; metabolic equivalent.

* Cut-off values for crude 25(OH)D (nmol/l) quintiles were 34·7, 44·2, 53·4 and 65·1. Cut-off values for season- and sex-stratified analysis could not be calculated.

**Figure 2.**
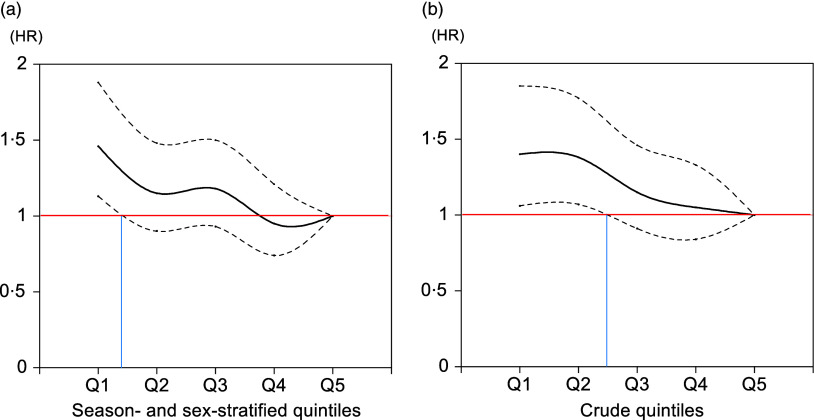
Cubic spline curves showing the multivariable-adjusted association between (a) season- and sex-stratified quintiles and (b) crude quintiles of plasma 25(OH)D with the hazard of all-cause mortality. The threshold of 25(OH)D concentration at which the lower bound of the 95 % confidence interval exceeded 1 was calculated to be 32·4 nmol/l when using season- and sex-stratified quintiles and 44·2 nmol/l when using crude quintiles. 25(OH)D, 25-hydroxyvitamin D.

All-cause mortality and its HR according to season- and sex-stratified and crude quintiles of 25(OH)D concentrations are shown in Table 2. Lower season- and sex-stratified quintiles were associated with higher hazards of mortality (*P*_for trend_ = 0·0015), with the first quintile having a higher HR (adjusted HR = 1·46, 95 % CI, 1·13, 1·88) than the highest quintile (reference). Lower crude quintiles were associated with higher hazards of mortality (adjusted *P*_for trend_ = 0·0027), with the first and second quintiles having higher HR (adjusted HR = 1·40, 95 % CI, 1·06, 1·85 and 1·38, 95 % CI, 1·07, 1·77) than the reference. Cubic spline curves for these associations are shown in Figure 2. In this graphical analysis, the threshold of 25(OH)D concentration at which the lower bound of the 95 % CI exceeded 1 was estimated to be 32·4 nmol/l when using season- and sex-stratified quintiles and 44·2 nmol/l when using crude quintiles. Similar analyses to those in Table 2 were conducted using sex-stratified and season-stratified quintiles for 25(OH)D groupings, with the results shown in online Supplementary Table 4.

**Table 2.** All-Cause mortality and hazard ratios (HR) according to different quintiles of plasma 25-hydroxyvitamin D (25(OH)D) concentrations

	Season- and sex-stratified 25(OH)D quintiles									*P* for trend
	Quintile 1		Quintile 2		Quintile 3		Quintile 4		Quintile 5	
	HR	95 % CI	HR	95 % CI	HR	95 % CI	HR	95 % CI		
Number of participants	1664		1661		1663		1658		1639	
Number of deaths	122		121		136		125		137	
Person-years (P-Y)	19 081		19 006		18 989		18 904		18 617	
Mortality rate (/1000P-Y)	6·4		6·4		7·2		6·6		7·4	
Unadjusted HR	0·86	0·67–1·10	0·86	0·67–1·09	0·97	0·76–1·23	0·89	0·70–1·14	1 (Ref)	0·2144
Age-adjusted HR	1·54	1·20–1·97	1·09	0·85–1·39	1·10	0·87–1·40	0·92	0·72–1·17	1 (Ref)	0·0007
Multivariable-adjusted HR^†^	1·46	1·13–1·88	1·15	0·90–1·48	1·18	0·93–1·50	0·95	0·74–1·21	1 (Ref)	0·0015

	Crude 25(OH)D quintiles^*^									*P* for trend
	Quintile 1		Quintile 2		Quintile 3		Quintile 4		Quintile 5	
Number of participants	1693		1594		1716		1640		1642	
Number of deaths	95		117		129		136		164	
Person-years (P-Y)	19 583		18 279		19 608		18 644		18 483	
Mortality rate (/1000P-Y)	4·9		6·4		6·6		7·3		8·9	
Unadjusted HR	0·53	0·41–0·69	0·71	0·56–0·90	0·73	0·58–0·92	0·82	0·65–1·02	1 (Ref)	<0·0001
Age-adjusted HR	0·91	0·70–1·17	0·88	0·69–1·11	0·81	0·65–1·02	0·87	0·69–1·09	1 (Ref)	0·3862
Multivariable-adjusted HR^‡^	1·40	1·06–1·85	1·38	1·07–1·77	1·15	0·91–1·46	1·05	0·84–1·33	1 (Ref)	0·0027

* Cut-off values for crude 25(OH)D (nmol/l) quintiles were 34·7, 44·2, 53·4 and 65·1. Cut-off values for season- and sex-stratified analysis could not be calculated.

† Adjusted for age, marital status, education, occupation, BMI, total physical activity, smoking, alcohol consumption and disease history.

‡ Adjusted for season score, sex, age, marital status, education, occupation, BMI, total physical activity, smoking, alcohol consumption and disease history.

All-cause mortality and its HR according to season- and sex-stratified and crude quintiles of 25(OH)D concentrations by sex are shown in Table 3. Lower season- and sex-stratified quintiles were associated with higher hazards of mortality (adjusted *P* for trend = 0·0222) in men, with the first quintile having a higher HR (adjusted HR = 1·38, 95 % CI, 1·02, 1·88) than the reference. Associations in women were similar to those in men, but not significant. Lower crude quintiles were associated with higher hazards of mortality (adjusted *P*_for trend_ = 0·0183) in men, with the second quintile having a higher HR (adjusted HR = 1·48, 95 % CI, 1·10, 1·99) than the reference. In women, lower crude quintiles were marginally associated with higher hazards of mortality (adjusted *P*_for trend_ = 0·0579), with the first quintile having a higher HR (adjusted HR = 1·71, 95 % CI, 1·02, 2·88) than the reference. Cubic spline curves for these associations by sex are shown in online Supplementary Figure 2. Based on season- and sex-stratified quintiles, the 25(OH)D concentration threshold at which the lower bound of the 95 % CI exceeded 1 was estimated to be 30·1 nmol/l for men and 29·1 nmol/l for women. When using crude quintiles, the corresponding thresholds were estimated to be 35·2 nmol/l for men and 29·4 nmol/l for women.

**Table 3.** All-Cause mortality and hazard ratios (HR) according to season- and sex-stratified quintiles of plasma 25-hydroxyvitamin D (25(OH)D) concentrations by sex

	Season- and sex-stratified 25(OH)D quintiles									*P* for trend
	Quintile 1		Quintile 2		Quintile 3		Quintile 4		Quintile 5	
	HR	95 % CI	HR	95 % CI	HR	95 % CI	HR	95 % CI		
Men										
Median 25(OH)D concentration	33·3		46·4		56·4		65·4		79·6	
Number of participants	724		722		730		725		715	
Number of deaths	84		80		93		87		95	
Person-years (P-Y)	8160		8109		8186		8093		7949	
Mortality rate (/1000P-Y)	10·3		9·9		11·4		10·7		12·0	
Unadjusted HR	0·85	0·63–1·14	0·82	0·61–1·10	0·95	0·71–1·26	0·89	0·67–1·20	1 (Ref)	0·2150
Age-adjusted HR	1·44	1·07–1·94	1·07	0·79–1·44	1·07	0·81–1·43	0·94	0·70–1·25	1 (Ref)	0·0179
Multivariable-adjusted HR^†^	1·38	1·02–1·88	1·16	0·86–1·58	1·17	0·87–1·56	0·98	0·73–1·31	1 (Ref)	0·0222
Women										
Median 25(OH)D concentration	25·7		36·4		44·4		52·4		66·4	
Number of participants	940		939		933		933		924	
Number of deaths	38		41		43		38		42	
Person-years (P-Y)	10 921		10 896		10 803		10 811		10 668	
Mortality rate (/1000P-Y)	3·5		3·8		4·0		3·5		3·9	
Unadjusted HR	0·88	0·56–1·36	0·94	0·61–1·45	1·00	0·66–1·53	0·88	0·57–1·37	1 (Ref)	0·6829
Age-adjusted HR	1·62	1·04–2·53	1·17	0·76–1·80	1·15	0·75–1·76	0·90	0·58–1·39	1 (Ref)	0·0218
Multivariable-adjusted HR^†^	1·47	0·94–2·31	1·15	0·74–1·77	1·12	0·73–1·72	0·91	0·58–1·41	1 (Ref)	0·0622

	Crude 25(OH)D quintiles^*^									*P* for trend
	Quintile 1		Quintile 2		Quintile 3		Quintile 4		Quintile 5	
Men										
Median 25(OH)D concentration	28·7		39·9		48·9		59·4		74·6	
Number of participants	427		537		663		866		1123	
Number of deaths	43		70		83		100		143	
Person-years (P-Y)	4839		6016		7437		9730		12 475	
Mortality rate (/1000P-Y)	8·9		11·6		11·2		10·3		11·5	
Unadjusted HR	0·76	0·54–1·07	1·00	0·75–1·33	0·96	0·73–1·26	0·89	0·69–1·15	1 (Ref)	0·3176
Age-adjusted HR	1·38	0·98–1·95	1·32	0·99–1·77	1·11	0·85–1·45	0·91	0·71–1·18	1 (Ref)	0·0115
Multivariable-adjusted HR^‡^	1·22	0·85–1·75	1·48	1·10–1·99	1·17	0·89–1·55	0·98	0·75–1·27	1 (Ref)	0·0183
Women										
Median 25(OH)D concentration	27·7		39·7		48·4		58·4		72·9	
Number of participants	1266		1057		1053		774		519	
Number of deaths	52		47		46		36		21	
Person-years (P-Y)	14 744		12 263		12 171		8914		6008	
Mortality rate (/1000P-Y)	3·5		3·8		3·8		4·0		3·5	
Unadjusted HR	0·99	0·60–1·65	1·09	0·65–1·81	1·07	0·64–1·79	1·16	0·68–1·98	1 (Ref)	0·7380
Age-adjusted HR	1·66	1·00–2·77	1·29	0·77–2·16	1·16	0·69–1·94	1·27	0·74–2·18	1 (Ref)	0·0571
Multivariable-adjusted HR^‡^	1·71	1·02–2·88	1·36	0·81–2·29	1·18	0·70–1·98	1·36	0·79–2·34	1 (Ref)	0·0579

* Cut-off values for crude 25(OH)D (nmol/l) quintiles were 34·7, 44·2, 53·4 and 65·1. Cut-off values for season- and sex-stratified analysis could not be calculated.

† Adjusted for age, marital status, education, occupation, BMI, total physical activity, smoking, alcohol consumption and disease history.

‡ Adjusted for season score, age, marital status, education, occupation, BMI, total physical activity, smoking, alcohol consumption and disease history.

All-cause mortality and HR according to season- and sex-stratified quintiles of plasma 25(OH)D concentrations by age group (< 65 *v*. ≥ 65 years) in men and women are shown in online Supplementary Table 5. The inverse association between them tended to be more robust in the age ≥ 65 years group (*P* for trend = 0·0449) in men and age < 65 years group (*P*_for trend_ = 0·0637) in women.

All-cause mortality and HR according to season- and sex-stratified quintiles of plasma 25(OH)D concentrations by season subgroup (season score 1–3 *v*. 4, 5) are shown in Table 4. Season- and sex-stratified 25(OH)D quintiles showed a dose-dependent association with mortality during seasons when plasma 25(OH)D concentrations were lower (adjusted HR = 1·55, 95 % CI: 1·10, 2·18, *P*_for trend_ = 0·0034), but not during seasons when concentrations were higher (adjusted HR = 1·31, 95 % CI: 0·90, 1·91, *P*_for trend_ = 0·2588).

**Table 4.** All-cause mortality and hazard ratios (HR) according to season- and sex-stratified and crude quintiles of plasma 25-hydroxyvitamin D (25(OH)D) concentrations by season group

	Season- and sex-stratified 25(OH)D quintiles									*P* for trend
	Quintile 1		Quintile 2		Quintile 3		Quintile 4		Quintile 5	
	HR	95 % CI	HR	95 % CI	HR	95 % CI	HR	95 % CI		
Season score^*^: 1–3										
Median 25(OH)D concentration	26·2		36·9		44·9		54·2		70·9	
Number of participants	906		899		902		917		896	
Number of deaths	68		74		79		71		78	
Person-years (P-Y)	10 504		10 361		10 383		10 555		10 304	
Mortality rate (/1000P-Y)	6·5		7·1		7·6		6·7		7·6	
Unadjusted HR	0·85	0·61–1·17	0·93	0·68–1·28	1·00	0·73–1·37	0·88	0·64–1·22	1 (Ref)	0·4581
Age-adjusted HR	1·61	1·15–2·24	1·22	0·89–1·68	1·16	0·85–1·59	0·91	0·66–1·25	1 (Ref)	0·0017
Multivariable-adjusted HR^‡^	1·55	1·10–2·18	1·31	0·94–1·81	1·23	0·90–1·69	0·97	0·70–1·34	1 (Ref)	0·0034
Season score^*^: 4, 5										
Median 25(OH)D concentration	31·1		41·9		49·4		58·4		77·4	
Number of participants	758		762		761		741		743	
Number of deaths	54		47		57		54		59	
Person-years (P-Y)	8577		8644		8606		8350		8313	
Mortality rate (/1000P-Y)	6·3		5·4		6·6		6·5		7·1	
Unadjusted HR	0·88	0·61–1·27	0·76	0·52–1·11	0·93	0·64–1·33	0·91	0·63–1·31	1 (Ref)	0·3008
Age-adjusted HR	1·43	0·99–2·08	0·92	0·63–1·36	1·02	0·71–1·47	0·94	0·65–1·36	1 (Ref)	0·1371
Multivariable-adjusted HR^‡^	1·31	0·90–1·91	0·94	0·64–1·39	1·11	0·77–1·61	0·91	0·62–1·32	1 (Ref)	0·2588

	Crude 25(OH)D quintiles^†^									*P* for trend
	Quintile 1		Quintile 2		Quintile 3		Quintile 4		Quintile 5	
Season score^*^: 1–3										
Median 25(OH)D concentration	27·5		39·7		48·4		59·2		73·1	
Number of participants	1119		950		910		846		695	
Number of deaths	66		74		79		72		79	
Person-years (P-Y)	13 045		10 983		10 452		9697		7930	
Mortality rate (/1000P-Y)	5·1		6·7		7·6		7·4		10·0	
Unadjusted HR	0·50	0·36–0·69	0·67	0·49–0·92	0·75	0·55–1·02	0·74	0·54–1·02	1 (Ref)	<0·0001
Age-adjusted HR	0·92	0·66–1·28	0·88	0·64–1·21	0·86	0·63–1·18	0·80	0·58–1·10	1 (Ref)	0·7988
Multivariable-adjusted HR^§^	1·34	0·94–1·92	1·31	0·94–1·83	1·15	0·84–1·59	0·91	0·66–1·25	1 (Ref)	0·0196
Season score^*^: 4, 5										
Median 25(OH)D concentration	28·7		39·7		48·8		58·7		74·6	
Number of participants	574		644		806		794		947	
Number of deaths	29		43		50		64		85	
Person-years (P-Y)	6538		7296		9155		8948		10 553	
Mortality rate (/1000P-Y)	4·4		5·9		5·5		7·2		8·1	
Unadjusted HR	0·54	0·36–0·83	0·72	0·50–1·04	0·67	0·47–0·95	0·89	0·64–1·23	1 (Ref)	0·0014
Age-adjusted HR	0·95	0·62–1·46	0·91	0·63–1·32	0·77	0·54–1·09	0·98	0·71–1·36	1 (Ref)	0·4797
Multivariable-adjusted HR^§^	1·44	0·92–2·26	1·44	0·97–2·13	1·12	0·78–1·61	1·29	0·92–1·79	1 (Ref)	0·0703

* (1) March and April (mean 25(OH)D, 42·6–42·8 nmol/l); (2) February and May (45·7–47·3 nmol/l); (3) June (49·0 nmol/l); (4) July, October, November, December and January (51·9–53·3 nmol/l) and (5) August and September (58·3–58·8 nmol/l).

† Cut-off values for crude 25(OH)D (nmol/l) quintiles were 34·7, 44·2, 53·4 and 65·1. Cut-off values for season- and sex-stratified analysis could not be calculated.

‡ Adjusted for sex, age, marital status, education, occupation, BMI, total physical activity, smoking, alcohol consumption and disease history.

§ Adjusted for season score, sex, age, marital status, education, occupation, BMI, total physical activity, smoking, alcohol consumption and disease history.

Mortality from and HR for cause-specific death from cancer, cardiovascular and cerebrovascular diseases and other causes according to season- and sex-stratified quintiles of 25(OH)D concentrations are shown in online Supplementary Table 6. Season- and sex-stratified quintiles of 25(OH)D concentrations were inversely associated with mortality from cancer (adjusted *P*_for trend_ = 0·0170) and other causes (adjusted *P*_for trend_ = 0·0257), with the first quintile having a higher HR (1·67, 95 % CI, 1·11, 2·50) for cancer. There was no association between 25(OH)D quintiles and mortality from cardiovascular and cerebrovascular diseases (adjusted *P*_for trend_ = 0·8891).

## Discussion

The present study is the first to report an inverse association between blood 25(OH)D concentrations and mortality in population-based cohorts in Japan. We found that all-cause mortality began to significantly increase at the lowest quintile (median = 28·2 nmol/l) when using season- and sex-stratified 25(OH)D groupings and at the second quintile (median = 39·7 nmol/l) when using crude 25(OH)D quintiles. This suggests that the risk threshold for vitamin D deficiency in relation to all-cause mortality is overestimated by approximately 10 nmol/l when using the crude blood 25(OH)D concentrations, particularly in men. A potential explanation for this is the reclassification of individuals who should have been in the first quintile into the second quintile, thereby elevating the HR in the second quintile. This reclassification could have resulted from seasonal variations in blood collection, which occurred more frequently in non-winter than winter seasons. Consequently, individuals with relatively low vitamin D levels were less likely to be classified as deficient when assessed solely based on crude 25(OH)D concentrations. In addition, vitamin D levels in men were much higher than in women, which may also have contributed to this discrepancy.

Several cohort studies published in the last decade have reported the risk threshold of blood 25(OH)D for all-cause mortality using a season variable (i.e. a variable representing the four seasons) as a covariate in multivariate analysis. Durazo-Arvizu *et al.* ^(8)^ reported a threshold of 40 nmol/l when comparing 25(OH)D concentrations by dividing them into increments of 10 nmol/l in a study of 15 099 USA individuals aged ≥ 20 years (mean age: 45 years; mean 25(OH)D concentration, 64 nmol/l). Zhu *et al.* ^(13)^ reported a threshold of 60 nmol/l, determined by the lower bound of the 95 % CI which exceeded 1 on a cubic spline curve in a study of 3946 Australians aged 25–84 years (mean age: 52·5 years; mean 25(OH)D concentration: 60·6 nmol/l). In two studies that used quartile groupings, Sun *et al.* ^(7)^ reported a threshold of 35 nmol/l (upper bound of the lowest quartile) in a study of 6613 Norwegians aged ≥ 20 years (mean age: 47·3 years; mean 25(OH)D concentration: 47·3 nmol/l), and Park *et al.* ^(11)^ reported a threshold of 55 nmol/l (upper bound of the third quartile) in 27 846 Koreans aged ≥ 20 years (mean age: 45·1 years; mean 25(OH)D concentration: 44·5 nmol/l). Two studies adopting the USA Institute of Medicine’s 25(OH)D classification (< 30, 30–49, 50–74, ≥ 75 nmol/l) reported a threshold of 50 nmol/l in a study of 11 022 NHANES III participants (mean age: 54·3 years; mean 25(OH)D concentration: 75 nmol/l)^(9)^ and a threshold of 50 nmol/l in a study of 18 797 Koreans aged ≥ 40 years (mean age 58·2 years; mean 25(OH)D concentration: 55·8 nmol/l)^(10)^. Finally, a large UK Biobank cohort study reported thresholds of 45–60 nmol/l. Compared with the results of previous studies, the estimated threshold of between 28·2 and 39·4 (medians of Quintile 1 and Quintile 2 using season- and sex-stratified 25(OH)D groupings) or 44·2 nmol/l (upper boundary of Quintile 2 using crude 25(OH)D grouping) in the present study (mean age: 59·9 years; mean 25(OH)D concentration: 50·1 nmol/l) lies around the midpoint or lower. This lower threshold may at least in part be attributed to the null association between 25(OH)D groups and mortality from cardiovascular and cerebrovascular diseases (shown in online Supplementary Table 6), probably due to low mortality from cardiovascular disease in Japan^(19)^.

Given that the COVID-19 pandemic, which began in 2020, may have influenced mortality rates, we assessed all-cause mortality and HR according to season- and sex-stratified quintiles of plasma 25(OH)D concentrations based on follow-up through 2019 (mean follow-up period: 7·4 years) (online Supplementary Table 7). The trends were generally similar to those observed in the overall analysis (Table 2), but the HR for the lowest quintile was 1·27 (95 % CI: 0·86, 1·87), which tended to be lower than that in the overall analysis (HR = 1·46). Thus, there may have been a potential interaction effect of COVID-19 on the association between blood 25(OH)D concentrations and mortality.

The 25(OH)D concentrations observed in the present study population should be compared with those of other Japanese populations to determine if they are representative. Two population-based studies of the general Japanese population reported prevalence rates of blood 25(OH)D concentration < 75 nmol/l of 72 % in men and 88 % in women (*n* 1683; mean age, 70·3 years; October–January)^(20)^ and 90 % in women (*n* 1211; mean age, 64·1 years; September–December)^(21)^. In the present study, the corresponding prevalence rates were 85 % in men and 96 % in women (data not shown), suggesting that prevalence rates in the present study were slightly higher than those reported in previous studies. This could be explained by the slightly younger study population in the present study relative to those in previous studies, as younger Japanese adults tend to have lower blood 25(OH)D concentrations^(16)^.

Strengths of the present study include the large sample size and demonstration that relying solely on statistical adjustments for key confounding factors such as season and sex may not be appropriate when analysing the strength of associations between 25(OH)D concentrations and mortality risk. The present study also has some limitations. First, blood 25(OH)D concentration was measured once at baseline, and its changes during the follow-up period were not evaluated. This may have resulted in a discrepancy in the classification of 25(OH)D concentrations. Second, we did not account for all confounding factors affecting the association between 25(OH)D concentration and mortality. Accordingly, there may be unknown confounders that could have influenced the observed associations. Third, we used the chemiluminescent immunoassay method to measure blood 25(OH)D concentrations. However, the chemiluminescent immunoassay method has been reported to yield lower vitamin D values compared to LC–MS/MS (the gold standard, approximately 10 %^(22,23)^), which may have led to an overestimation of vitamin D deficiency. Therefore, the cut-off value of blood 25(OH)D concentration for mortality may be slightly lower than that suggested in the present study. Finally, generalisation of our results should be approached with caution. Blood collection was conducted mostly in non-winter seasons, which may have led to false vitamin D sufficiency and introduced classification discrepancies. Given the marked sex difference in 25(OH)D concentrations, these types of discrepancies may be less likely in settings where blood collection occurs during seasons with low sunlight exposure and in populations with smaller sex differences.

The present study demonstrated that using season- and sex-stratified quintiles of blood 25(OH)D concentrations effectively removed the confounding effects of season of blood collection and sex, which are important factors influencing the relationship between 25(OH)D concentrations and disease occurrence. This approach enabled the identification of a 25(OH)D concentration associated with mortality that is less affected by these factors. However, the observed differences between season- and sex-stratified quintiles and crude quintiles suggest that the threshold may vary depending on season or sex. While no sex-specific differences in the threshold were observed, a dose-dependent increase in mortality risk was observed as 25(OH)D concentrations declined among participants whose vitamin D levels were measured during seasons when 25(OH)D concentrations tend to be lower, rather than the risk being elevated only in the lowest quintile. These findings suggest that the association between vitamin D levels and mortality may differ by season.

### Conclusions

Blood 25(OH)D concentrations are inversely associated with all-cause mortality in middle-aged and older Japanese people, and the risk threshold for vitamin D deficiency associated with mortality based on season- and sex-stratified 25(H)D groupings ranged from 28·2 to 39·4, with no sex difference. Crude blood 25(OH)D concentration may modulate the estimated risk threshold for vitamin D deficiency associated with mortality. Further studies on whether the threshold of the association between blood 25(OH)D concentrations and mortality varies by season are warranted.

## Supporting information

Kitamura et al. supplementary material 1 Kitamura et al. supplementary material

Kitamura et al. supplementary material 2 Kitamura et al. supplementary material

Kitamura et al. supplementary material 3 Kitamura et al. supplementary material

## Supplementary material

For supplementary material/s referred to in this article, please visit https://doi.org/10.1017/S0007114525105308
