# Supplementary material for: Plasma 25-hydroxyvitamin D concentrations, vitamin D deficiency and mortality in community-dwelling Japanese adults
Source: Br J Nutr. 2025 Oct 9;134(8):634–44. doi: 10.1017/S0007114525105308 (PMC12722008; doi:10.1017/S0007114525105308)
Supplement: Kitamura et al. supplementary material 1 — Kitamura et al. supplementary material [file S0007114525105308sup001.docx]

Supplementary Table 1. Codes and explanations for demographics, lifestyles, body mass index (BMI), and disease history

Variables

Marital status (a) married, (b) never married, and (c) divorced, separated, or bereaved (converted to dummy variables in the multivariate analysis)

Education level (1) junior high school, (2) high school, (3) junior or vocational college, and (4) university graduates

Occupation (a) office work and sales/service work, (b) professional/management, (c) manual (security, farming/forestry/fishery, transportation, and labor services), and (d) no job/others (converted to dummy variables in the multivariate analysis)

Smoking (1) non-smoker, (2) past smoker, (3) 1–20 cigarettes/day, and (4) ≥20 cigarettes/day

Alcohol consumption (1) 0, (2) 1–149, (3) 150–299, (4) 300–449, and (5) ≥450 g ethanol/week

Total physical activity Intensity of activity: sitting (1.3 METs), standing (2.0 METs), walking (3.0 METs), strenuous work (6.0 METs), walking slowly (2.8 METs), walking quickly (4.0 METs), light to moderate exercise (3.0 METs), strenuous exercise (6.0 METs), sleep (0.9 METs), and other activities (1.3 METs)

History of a disease (0) No and (1) Yes

Supplementary Table 2. Prevalence of low plasma 25(OH)D concentrations (<50 nmol/L) according to season and sex at baseline

Prevalence of 25(OH)D <50 nmol/L

Season score

1 (March and April) 441/658 (67.0%)

2 (February and May) 906/1433 (63.2%)

3 (June) 1318/2429 (54.3%)

4 (July, October, November, December, and January) 1542/3224 (47.8%)

5 (August and September) 188/541 (34.8%)

Sex

Men 1356/3616 (37.5%)

Women 3039/4669 (65.1%)

Supplementary Table 3. Agreement between season- and sex-stratified quintiles and crude quintiles of plasma 25-hydroxyvitamin D (25[OH]D) concentrations

Season- and sex-stratified 25(OH)D quintiles

Crude 25(OH)D quintiles Quintile 1 Quintile 2 Quintile 3 Quintile 4 Quintile 5

Quintile 1 1332 343 18 0 0

Quintile 2 297 823 450 24 0

Quintile 3 32 410 716 551 7

Quintile 4 3 85 439 699 414

Quintile 5 0 0 40 384 1218

Weighted κ coefficient was 0.72 (95% CI: 0.71–0.73).

Supplementary Table 4. All-cause mortality and hazard ratios (HRs) according to sex-stratified and season-stratified quintiles of plasma 25-hydroxyvitamin D (25[OH]D) concentrations.

Sex-stratified 25(OH)D quintiles P for trend

Quintile 1 Quintile 2 Quintile 3 Quintile 4 Quintile 5

Number of participants 1659 1645 1696 1637 1648

Number of deaths 122 128 139 123 129

Person-years (P-Y) 19047 18835 19345 18673 18697

Mortality rate (/1000P-Y) 6.4 6.8 7.2 6.6 6.9

Unadjusted HR 0.91 (0.71-1.17) 0.97 (0.76-1.24) 1.03 (0.81-1.31) 0.95 (0.74-1.21) 1 (Ref) 0.5716

Adjusted HR^*^ 1.56 (1.20-2.02) 1.27 (0.99-1.62) 1.20 (0.95-1.53) 1.01 (0.79-1.30) 1 (Ref) 0.0003

Season-stratified 25(OH)D quintiles^*^ P for trend

Quintile 1 Quintile 2 Quintile 3 Quintile 4 Quintile 5

Number of participants 1638 1691 1664 1675 1617

Number of deaths 93 125 108 153 162

Person-years (P-Y) 18886 19395 19089 19000 18227

Mortality rate (/1000P-Y) 4.9 6.4 5.7 8.1 8.9

Unadjusted HR 0.55 (0.42-0.70) 0.71 (0.57-0.90) 0.63 (0.49-0.80) 0.90 (0.72-1.12) 1 (Ref) <0.0001

Adjusted HR^†^ 1.53 (1.16-2.02) 1.41 (1.10-1.80) 1.04 (0.81-1.34) 1.18 (0.95-1.48) 1 (Ref) 0.0013

*Note:* 95% confidence intervals are shown in parentheses

^*^Adjusted for season score, age, marital status, education, occupation, BMI, total physical activity, smoking, alcohol consumption, and disease history

^†^Adjusted for sex, age, marital status, education, occupation, BMI, total physical activity, smoking, alcohol consumption, and disease history

Supplementary Table 5. All-cause mortality and hazard ratios (HRs) according to season- and sex-stratified and crude quintiles of plasma 25-hydroxyvitamin D (25[OH]D) concentrations by age group in men and women.

Season- and sex-stratified 25(OH)D quintiles P for trend

Quintile 1 Quintile 2 Quintile 3 Quintile 4 Quintile 5

Men

Age <65 years

Number of participants 565 479 436 390 384

Number of death 36 28 32 25 29

Person-years (P-Y) 6478 5481 4990 4432 4331

Mortality rate (/1000P-Y) 5.6 5.1 6.4 5.6 6.7

Unadjusted HR 0.82 (0.50-1.34) 0.75 (0.45-1.27) 0.95 (0.58-1.57) 0.84 (0.49-1.43) 1 (ref) 0.3847

Adjusted HR^*^ 1.29 (0.77-2.16) 1.14 (0.67-1.95) 1.22 (0.73-2.05) 1.04 (0.60-1.79) 1 (ref) 0.3216

Age ≥65 years

Number of participants 159 243 294 335 331

Number of death 48 52 61 62 66

Person-years (P-Y) 1682 2628 3197 3661 3618

Mortality rate (/1000P-Y) 28.5 19.8 19.1 16.9 18.2

Unadjusted HR 1.58 (1.09-2.29) 1.08 (0.75-1.55) 1.05 (0.74-1.48) 0.92 (0.65-1.31) 1 (ref) 0.0254

Adjusted HR^*^ 1.44 (0.98-2.12) 1.16 (0.80-1.68) 1.11 (0.78-1.58) 0.95 (0.67-1.35) 1 (ref) 0.0449

Women

Age <65 years

Number of participants 788 645 617 560 552

Number of death 22 14 8 14 9

Person-years (P-Y) 9169 7516 7214 6493 6419

Mortality rate (/1000P-Y) 2.4 1.9 1.1 2.2 1.4

Unadjusted HR 1.69 (0.78-3.67) 1.30 (0.56-3.01) 0.78 (0.30-2.02) 1.52 (0.66-3.52) 1 (ref) 0.2424

Adjusted HR^*^ 2.22 (1.00-4.91) 1.52 (0.65-3.54) 0.82 (0.32-2.13) 1.59 (0.69-3.70) 1 (ref) 0.0637

Age ≥65 years

Number of participants 152 294 316 373 372

Number of death 16 27 35 24 33

Person-years (P-Y) 1753 3381 3589 4318 4249

Mortality rate (/1000P-Y) 9.1 8.0 9.8 5.6 7.8

Unadjusted HR 1.16 (0.64-2.11) 1.02 (0.61-1.69) 1.26 (0.78-2.02) 0.71 (0.42-1.20) 1 (ref) 0.3349

Adjusted HR^*^ 1.06 (0.57-1.95) 1.01 (0.60-1.70) 1.26 (0.78-2.04) 0.72 (0.42-1.22) 1 (ref) 0.4597

*Note:* 95% confidence intervals are shown in parentheses

^*^Adjusted for age, marital status, education, occupation, BMI, total physical activity, smoking, alcohol consumption, and disease history

Supplementary Table 6. Cause-specific mortality and hazard ratios (HRs) according to season- and sex-stratified quintiles of plasma 25-hydroxyvitamin D (25[OH]D) concentrations.

Season- and sex-stratified 25(OH)D quintiles P for trend

Quintile 1 Quintile 2 Quintile 3 Quintile 4 Quintile 5

Cancer

Number of participants 1664 1661 1663 1658 1639

Number of deaths 52 41 40 43 50

Person-years (P-Y) 17156 17083 17083 16990 16742

Mortality rate (/1000P-Y) 3.0 2.4 2.3 2.5 3.0

Unadjusted HR 1.01 (0.69-1.49) 0.80 (0.53-1.21) 0.78 (0.52-1.18) 0.84 (0.56-1.27) 1 (ref) 0.9616

Adjusted HR^*^ 1.67 (1.11-2.50) 1.08 (0.71-1.64) 0.95 (0.63-1.45) 0.92 (0.61-1.38) 1 (ref) 0.0170

Cardiovascular and cerebrovascular diseases

Number of participants 1664 1661 1663 1658 1639

Number of deaths 16 19 32 30 22

Person-years (P-Y) 17156 17083 17083 16990 16742

Mortality rate (/1000P-Y) 0.9 1.1 1.9 1.8 1.3

Unadjusted HR 0.70 (0.37-1.34) 0.84 (0.45-1.54) 1.41 (0.82-2.43) 1.33 (0.77-2.31) 1 (ref) 0.1057

Adjusted HR^*^ 1.05 (0.54-2.04) 1.01 (0.54-1.88) 1.59 (0.92-2.75) 1.35 (0.78-2.35) 1 (ref) 0.8891

Other causes

Number of participants 1664 1661 1663 1658 1639

Number of deaths 28 35 35 22 34

Person-years (P-Y) 17156 17083 17083 16990 16742

Mortality rate (/1000P-Y) 1.6 2.0 2.0 1.3 2.0

Unadjusted HR 0.79 (0.48-1.30) 0.99 (0.62-1.59) 1.00 (0.62-1.60) 0.63 (0.37-1.08) 1 (ref) 0.9095

Adjusted HR^*^ 1.39 (0.83-2.34) 1.40 (0.87-2.27) 1.26 (0.78-2.03) 0.68 (0.40-1.17) 1 (ref) 0.0257

*Note:* 95% confidence intervals are shown in parentheses

^*^Adjusted for age, marital status, education, occupation, BMI, total physical activity, smoking, alcohol consumption, and disease history

Supplementary Table 7. All-cause mortality and hazard ratios (HRs) according to season- and sex-stratified quintiles of plasma 25-hydroxyvitamin D (25[OH]D) concentrations, based on follow-up through 2019 (the year prior to the COVID-19 pandemic)

Season- and sex-stratified 25(OH)D quintiles P for trend

Quintile 1 Quintile 2 Quintile 3 Quintile 4 Quintile 5

Number of participants 1664 1661 1663 1658 1639

Number of death 50 57 55 63 60

Person-years (P-Y) 12354 12323 12318 12228 12045

Mortality rate (/1000P-Y) 4.0 4.6 4.5 5.2 5.0

Unadjusted HR 0.81 (0.55-1.17) 0.92 (0.64-1.32) 0.89 (0.62-1.29) 1.03 (0.72-1.47) 1 (ref) 0.2031

Adjusted HR^*^ 1.27 (0.86-1.87) 1.20 (0.83-1.74) 1.07 (0.74-1.55) 1.11 (0.78-1.58) 1 (ref) 0.2092

*Note:* 95% confidence intervals are shown in parentheses

^*^Adjusted for age, marital status, education, occupation, BMI, total physical activity, smoking, alcohol consumption, and disease history
