# Supplementary material for: Plasma 25-hydroxyvitamin D concentrations, vitamin D deficiency and mortality in community-dwelling Japanese adults
Source: Br J Nutr. 2025 Oct 9;134(8):634–44. doi: 10.1017/S0007114525105308 (PMC12722008; doi:10.1017/S0007114525105308)
Supplement: Kitamura et al. supplementary material 2 — Kitamura et al. supplementary material [file S0007114525105308sup002.pptx]

## Slide 1
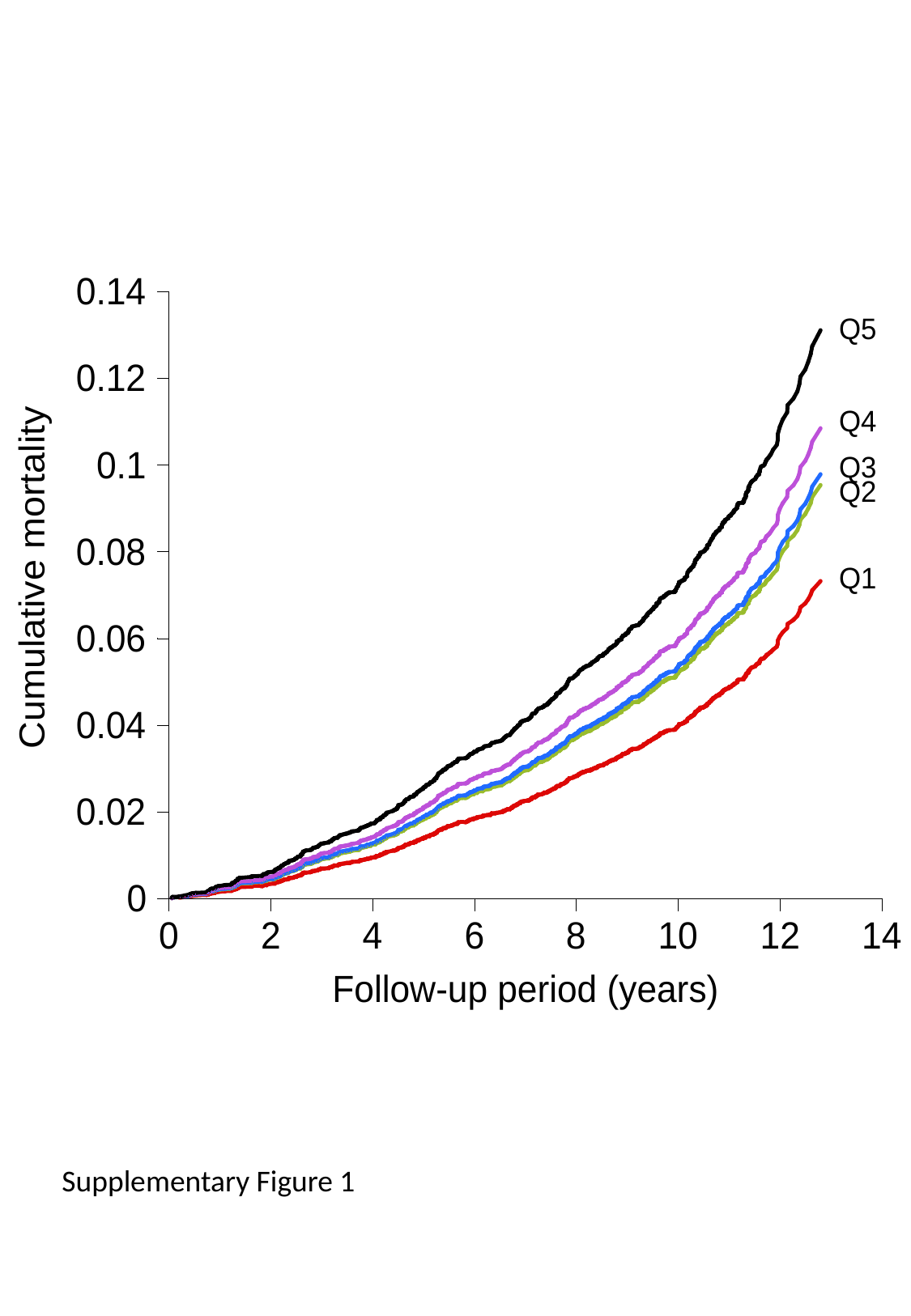

Supplementary Figure 1

## Slide 2
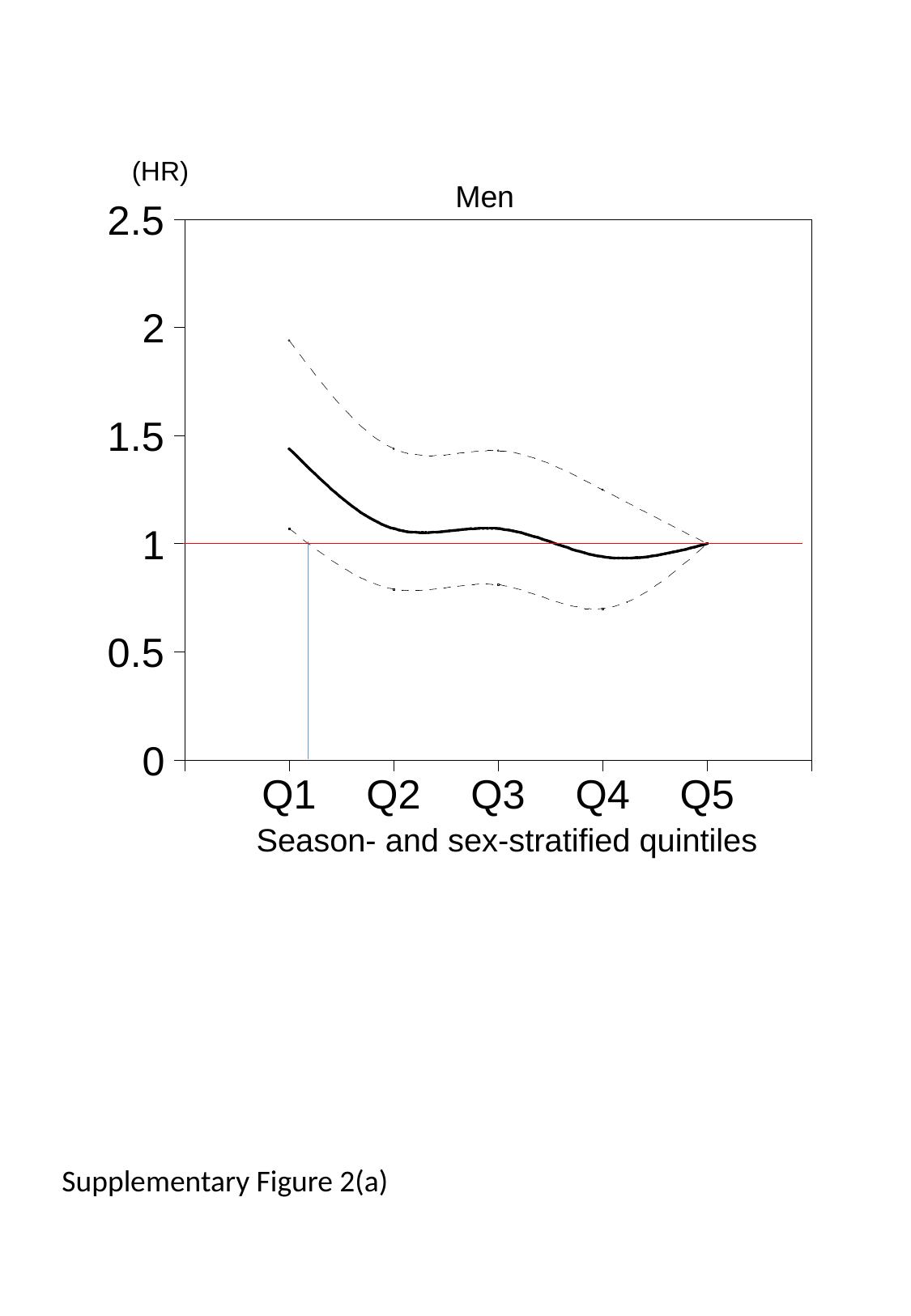

Men
Supplementary Figure 2(a)

## Slide 3
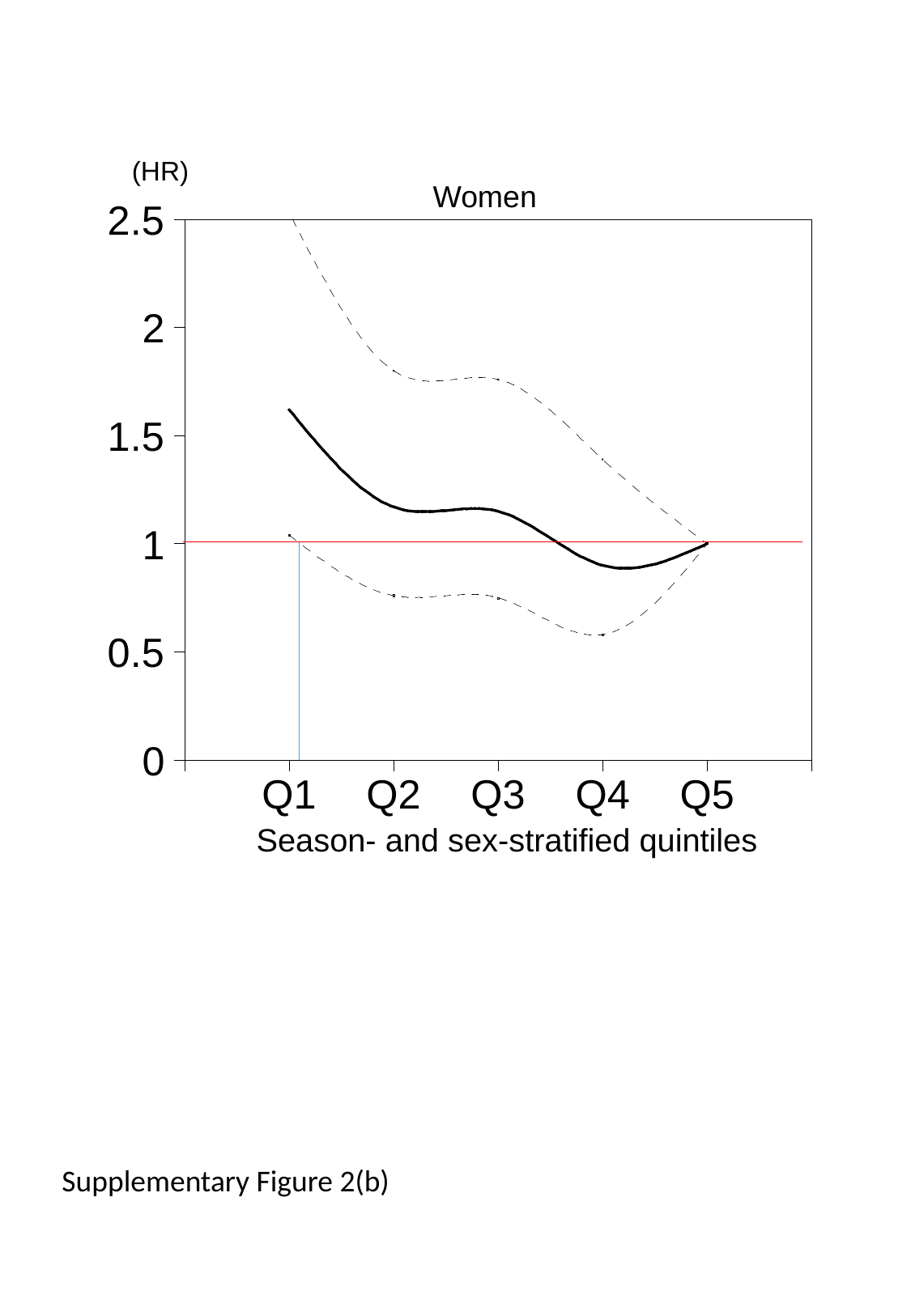

Women
Supplementary Figure 2(b)

## Slide 4
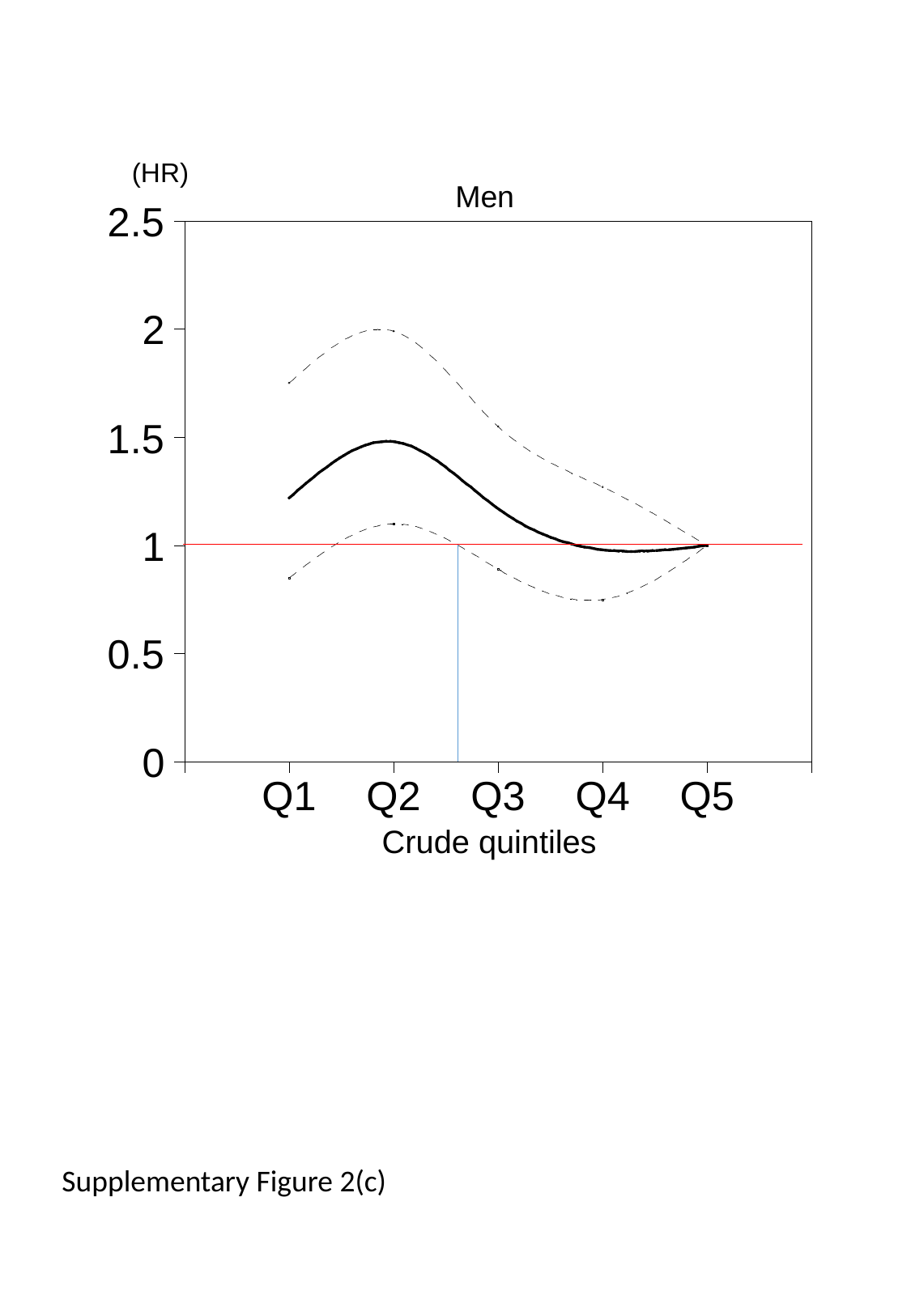

Men
Supplementary Figure 2(c)

## Slide 5
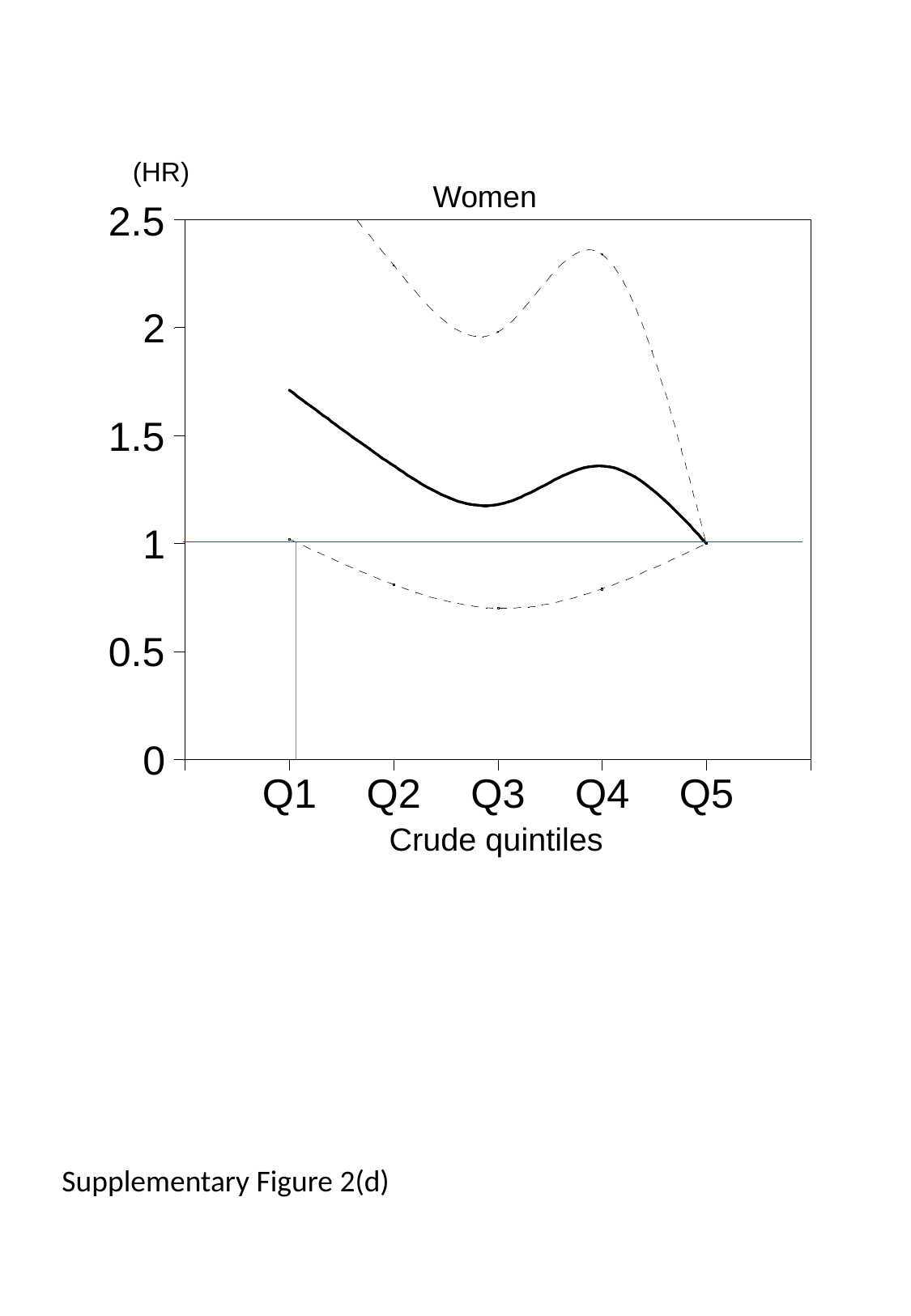

Women
Supplementary Figure 2(d)
