# Supplementary material for: Plasma 25-hydroxyvitamin D concentrations, vitamin D deficiency and mortality in community-dwelling Japanese adults
Source: Br J Nutr. 2025 Oct 9;134(8):634–44. doi: 10.1017/S0007114525105308 (PMC12722008; doi:10.1017/S0007114525105308)
Supplement: Kitamura et al. supplementary material 3 — Kitamura et al. supplementary material [file S0007114525105308sup003.docx]

**Supplementary Figure caption**

Supplementary Figure 1. Cumulative all-cause mortality, as determined by the Kaplan–Meier method, stratified by crude quintiles of 25(OH)D concentrations.

Supplementary Figure 2. Cubic spline curves showing the multivariable-adjusted association between season- and sex-stratified quintiles of plasma 25(OH)D concentration and the hazard of all-cause mortality in men (a) and women (b), and between crude quintiles of plasma 25(OH)D concentration and the hazard of all-cause mortality in men (c) and women (d). The threshold of 25(OH)D concentration at which the lower bound of the 95% confidence interval exceeded 1 was calculated to be 30.1 for men (a), 29.1 for women (b), 35.2 for men (c), and 29.4 nmol/L for women (d).
